# Structural Similarity-Induced Inter-Component Interaction in Silicone Polymer-Based Composite Sunscreen Film for Enhanced UV Protection

**DOI:** 10.3390/polym16233317

**Published:** 2024-11-27

**Authors:** Yuyan Chen, Hanwen Xu, Yuhang Liu, Qiuting Fu, Pingling Zhang, Jie Zhou, Hongyu Dong, Xiaodong Yan

**Affiliations:** 1 Research & Innovation Center, Proya Cosmetics Co., Ltd., Hangzhou 310023, China; chenyuyan@proya.com (Y.C.); liuyuhang@proya.com (Y.L.); fuqiuting@proya.com (Q.F.); zhangpingling@proya.com (P.Z.); zhoujie74@proya.com (J.Z.); donghongyu@proya.com (H.D.); 2 Key Laboratory of Synthetic and Biological Colloids, Ministry of Education, School of Chemical & Material Engineering, Jiangnan University, Wuxi 214122, China

**Keywords:** sunscreen, film-forming agent, polymer composites, ultraviolet protection

## Abstract

Film-forming agents are key ingredients in achieving long-lasting and effective sun protection by sunscreens. However, studies on the synergistic effects of film-forming agents with different properties as well as the interaction between film-forming agents and powders are scarce, restricting the development of sunscreens with strong ultraviolet (UV)-shielding effects. Herein, we innovatively adopt polysiloxane-15 as the soft film, trimethylsiloxysilicate as the hard film, and vinyl dimethicone/methicone silsesquioxane crosspolymer as the functional powder to construct a co-assembled sunscreen film, and we investigate the property-enhancing effects of the sunscreen film as well as the interaction between the silicone polymer-based film-forming agents and functional powder therein. The results show that structural similarity is essential to generating film-forming agent–powder interactions, which primarily enhance the Si−O bond binding energy, thereby enhancing the lasting protection effect of sunscreens. In addition, the inter-component interaction of the co-assembled sunscreen film inhibits the agglomeration of sunscreen paste to facilitate the formation of a homogeneous film, endowing the sunscreen with excellent UV protection abilities, with the sun protection factor (SPF) and protection factor of UVA (PFA) values increased by 61.58 and 43.84%, respectively. This work offers novel insights into the optimization of film-forming agent properties and the development of durable and efficient sunscreens.

## 1. Introduction

The increased ultraviolet (UV) intensity caused by continuous destruction of the ozone layer has led to a series of problems, such as accelerated melanin synthesis, collagen degradation, and increased skin sensitivity [1,2,3,4,5], which has motivated consumers to increase their awareness of sun protection and development of the sunscreen market [6,7,8,9,10,11]. It is generally accepted that sunscreen products with high sun protection factor (SPF) provide better UV shielding [12,13,14], but this does not mean that a high level of protection can be achieved on a long-lasting basis. For instance, external friction and perspiration can disrupt the distribution of sunscreen agents, which in turn reduces the effectiveness of sunscreens [15,16,17,18,19]. Moreover, the film-forming behavior of the sunscreen film can also alter the distribution of sunscreen agents, thereby directly affecting the sun protection value of the sunscreen [20,21]. Therefore, an effective strategy urgently needs to be developed to enhance the long-lasting and efficient UV-shielding effect of sunscreens.

The sunscreen film is mainly shaped by the film-forming agent in the formula, and many studies have confirmed that the sunscreen film is the key component in achieving durable and efficient sun protection by sunscreen [22,23,24,25,26,27,28,29,30,31]. For example, Infante et al. found that the addition of film-forming agent PEG-75 lanolin was able to achieve better sun protection without increasing the amount of UV filters, and in vivo tests showed that the SPF value of a sample containing the film-forming agent was increased by 53.2% compared with a blank sample, which confirmed the sun protection enhancement by the film-forming agent [23]. Keshavarzi et al. evaluated the sweat resistance of sunscreens by varying the concentration of hydrophobic film-forming agents, which showed that medium levels of film-forming agents inhibited the redistribution of sunscreens when encountered with sweat and higher levels of film-forming agents reduced the amount of sunscreen washed off by sweat [26]. Although numerous efforts have been devoted to the study of film-forming agents, focusing only on a single component, the synergistic effects of film-forming agents with different properties are rarely investigated, especially the intrinsic interaction between film-forming agents and other components, resulting in limited long-lasting exertion of strong UV-shielding effects when applied in sunscreen formulations. The development of composite sunscreen films with excellent film-forming properties is required to meet the growing consumer demand for sunscreens.

Herein, we adopted a co-assembly strategy to combine rigid trimethylsiloxysilicate and flexible polysiloxane-15 film-forming agents to achieve a balance of film-forming strength and toughness, and we innovatively screened the specific functional powder vinyl dimethicone/methicone silsesquioxane crosspolymer (VDSC) to further reinforce the sunscreen film. The anti-stretching, anti-migration, anti-friction, sunscreen enhancement, and water resistance properties of different formulations were compared and tested. In addition, a combination of testing and characterization was employed to analyze the mechanisms of action of the film-forming agents and powder in the co-assembled sunscreen film.

## 2. Materials and Methods

### 2.1. Materials

All reagents were purchased from raw material vendors and used directly without further purification. The ingredients used in the experiment are listed according to the International Nomenclature of Cosmetic Ingredients: water (Wahaha, Hangzhou, China); propanediol (Meijingrong Chemistry, Suzhou, China); 1,2-hexanediol (Shinsung Materials, Seoul, Republic of Korea); citric acid (Seven Star Lemon Technology, Linyi, China); sodium citrate (Xilong Scientific, Shantou, China); polysiloxane-15 (DSM, Maastricht, The Netherlands); trimethylsiloxysilicate (Wacker, Munich, Germany); vinyl dimethicone/methicone silsesquioxane crosspolymer (ShinEtsu, Tokyo, Japan); ethylhexyl methoxycinnamate (DSM, Maastricht, The Netherlands); ethylhexyl salicylate (DSM, Maastricht, The Netherlands); octocrylene (Symrise, Holzminden, Germany); titanium dioxide (Hope-Tec Biotechnology, Shanghai, China); zinc oxide (Hope-Tec Biotechnology, Shanghai, China); cyclopentasiloxane (Momentive, Wilton, CT, USA); dimethicone (Momentive, Wilton, CT, USA); PEG-9 polydimethylsiloxyethyl dimethicone (ShinEtsu, Tokyo, Japan); PEG-10 dimethicone (ShinEtsu, Tokyo, Japan); tocopheryl acetate (NHU, Shaoxing, China); isododecane (Goodlight, Qingyuan, China); alcohol (Ante food, Suzhou, China); phenoxyethanol (Ashland, Covington, KY, USA); ethylhexylglycerin (Ashland, Covington, KY, USA); dextrin palmitate (Chiba Flour Milling, Chiba, Japan); VP/eicosene copolymer (Ashland, Covington, KY, USA); and talc (Ritian Nanotechnology, Dandong, China).

### 2.2. Preparation of Sunscreen Samples

The co-assembled sunscreen film was composed of three silicone polymer-based components: polysiloxane-15, trimethylsiloxysilicate, and VDSC, in which polysiloxane-15 served as the soft film, trimethylsiloxysilicate served as the hard film, and VDSC served as the functional powder. The three components possessed similar silicone-oxygen bonded structures, and the corresponding structural formulae are shown schematically in Figures S1–S3.

The formulation of the sunscreen sample containing the co-assembled sunscreen film is shown in Table 1. The specific process was as follows: Firstly, phase A and phase B were heated to 78~80 °C and homogenized using a blender (RW20, IKA, Staufen, Germany). Then, phase A was slowly transferred to phase B, stirred to homogeneity, and emulsified using a homogenizer (T25, IKA, Staufen, Germany) at a homogenization speed of 6000 rpm for 3 min. Next, the temperature was cooled down to below 40 °C, phase C was added, and the mixture was homogenized continuously for 2 min, ultimately obtaining the sunscreen containing the co-assembled sunscreen film, labeled as CA.

Preparation of control samples: Based on the above method and the formulation shown in Table 1, polysiloxane-15, trimethylsiloxysilicate, and VDSC were removed to obtain the blank control sample without the co-assembled sunscreen film, labeled as BC; polysiloxane-15 was removed to obtain the sunscreen sample with a non-soft film, labeled as NS; trimethylsiloxysilicate was removed to obtain the non-hard-film sample, labeled as NH; and VDSC was removed to obtain the sample (labeled as NP) without powder. In addition, polysiloxane-15 was replaced with dextrin palmitate to obtain the sunscreen sample with replacement of the soft film, labeled as RS; trimethylsiloxysilicate was replaced with VP/eicosene copolymer to obtain the sunscreen sample with replacement of the hard film, labeled as RH; and VDSC was replaced with talc to obtain the sunscreen sample with replacement of the functional powder, labeled as RP.

### 2.3. Physical Characterization

#### 2.3.1. Micromorphological Characterization of the Co-Assembled Sunscreen Film

Scanning electron microscopy (SEM, Apreo S HiVac, Thermo Scientific, Waltham, MA, USA) was used to analyze the film-forming toughness with different combinations of components in the co-assembled sunscreen film. The raw material trimethylsiloxysilicate was recorded as sample A; polysiloxane-15 as sample B; polysiloxane-15 and trimethylsiloxysilicate mixed in a 2:3 mass ratio as sample C; and polysiloxane-15, trimethylsiloxysilicate, and VDSC in a 2:3:2 mix as sample D. The samples were prepared by dispersing A, B, C, and D into isododecane (volatile solvent), taking 50 μL of the samples and adding it dropwise on the conductive adhesive substrates. The conductive adhesive substrates loaded with samples were then transferred to a vacuum-drying oven at 40 °C for 24 h to form a membrane. Finally, all samples were stretched at the same strength to observe the microscopic film structure after stretching. Moreover, SEM images of VDSC and talc were also observed.

#### 2.3.2. Fourier-Transform Infrared Spectroscopy and X-Ray Photoelectron Spectroscopy Measurements

Fourier-transform infrared (FTIR, IRPrestige-21, Shimadzu, Kyoto, Japan) spectroscopy measurements were performed on VDSC, talc, and the film-forming agents (polysiloxane-15 and trimethylsiloxysilicate in a 2:3 mass ratio). To investigate the interaction between the powder and the film-forming agents, the powders after mixing and treatment with the film-forming agents were also tested. The treatment process of VDSC and talc was similar to the preparation process of the sunscreen samples, specifically, the powders and film-forming agent solutions were mixed according to a mass ratio of 2:5 and heated to 78~80 °C under stirring to create homogeneous dispersions. Then, the powders were homogenized at 6000 rpm for 3 min and centrifuged when the temperature was cooled to below 40 °C. Subsequently, the powders obtained by centrifugation were washed with deionized water and ethanol and dried at 60 °C in an oven. Furthermore, X-ray photoelectron spectroscopy (XPS, Thermo Scientific K-Alpha, Waltham, MA, USA) tests were performed on VDSC and film-forming agent-treated VDSC. All XPS spectra were calibrated using the C 1s signal at 284.8 eV.

#### 2.3.3. Optical Profilometer and Contact Angle Measurements

The film-forming uniformity of each sunscreen sample after film formation was assessed using an optical profilometer (NT9100, Veeco, Plainview, NY, USA). Firstly, the profilometer test was carried out by selecting a 1 mm × 1 mm area on the blank pigskin surface, thus serving as the profile mapping prior to the application of sunscreen samples. Then, 5 μL of the treated sunscreen samples was added dropwise to the selected area using a pipette. After the samples were completely dry, the profilometer test was again performed on the area. The wettability of VDSC and talc to the film-forming agent solutions was determined using an optical contact angle meter (OCA15EC, Dataphysics, Stuttgart, Germany), and the powders were pressed at 8 MPa for 5 min.

### 2.4. Performance Measurement

#### 2.4.1. Anti-Stretching Test

The sunscreen samples of 0.25 g were uniformly coated on the elastic silicone rubber belt with an area of 2 cm × 4 cm, and then left for 2.5 h to allow the sunscreens to form films. The initial condition of the film layer before stretching was first recorded using an optical microscope (MXB-2016Z, Hirox, Tokyo, Japan). After that, a unidirectional axial force was applied to all samples, which were stretched laterally to a length of 7.4~7.5 cm (deformation rate of about 85%) and fixed on the carrier stage with a G-clamp, observing whether holes and cracks appeared in the sunscreen film during stretching, thus evaluating the film-forming toughness of each sunscreen sample.

#### 2.4.2. Anti-Migrating Test

A 0.1 g aliquot of the sunscreen formulation samples BC, CA, and NP was added dropwise onto a polymethylmethacrylate (PMMA) plate and then spread evenly with a spatula. The distribution of the coatings during the evaporation of samples (film formation) was observed and recorded every 15 min, thus evaluating the anti-migrating property of the co-assembled sunscreen film.

#### 2.4.3. Anti-Rubbing Test

The formulation samples were evenly applied to the inner side of the arm with a loading of about 2 mg/cm^2^ and an application area of 2.5 cm × 2.5 cm. Subsequently, the area of the applied sample was rubbed with a paper towel loaded with an iron block (gravity of about 1.4 N). UV LOOK (the sunscreen application area behaves as black) was used to observe the scratches on the edge of the application area after rubbing and the residue on the surface of the paper towel, thereby evaluating the rubbing resistance of the samples.

#### 2.4.4. In Vitro Sun Protection Value Test

The method for in vitro testing of the sun protection value was based on the ISO 24443:2021 standard [32]. Specifically, samples CA, BC, NS, NH, and NP were added dropwise to a HD6-type PMMA plate (5 cm × 5 cm) with a loading of 1.3 mg/cm^2^, after which the samples were pushed away with a finger wearing a finger cuff in a circular motion to uniformly spread the samples on the PMMA plate. Finally, the PMMA panels coated with samples were dried at room temperature without direct sunlight for 30 min, and the SPF and protection factor of UVA (PFA) were determined using a UV transmittance analyzer (UV2000, Labsphere, North Charleston, SC, USA). Four PMMA plates were prepared for each sample and the average value was recorded as the initial sun protection value of the sample. In addition, the PMMA panels after testing were immersed in a water bath at 35 °C under low-speed agitation for 40 min, and then the sun protection value of the dried samples was tested and recorded as the after-bath sun protection value. The differences in the formulations of sunscreens CA, BC, NS, NH, and NP were variations in the three components, polysiloxane-15, trimethylsiloxysilicate, and VDSC (as shown in Table 2), while the other components were consistent with Table 1.

## 3. Results and Discussion

### 3.1. Micromorphological Analysis of the Co-Assembled Sunscreen Film

**Figure 1:**
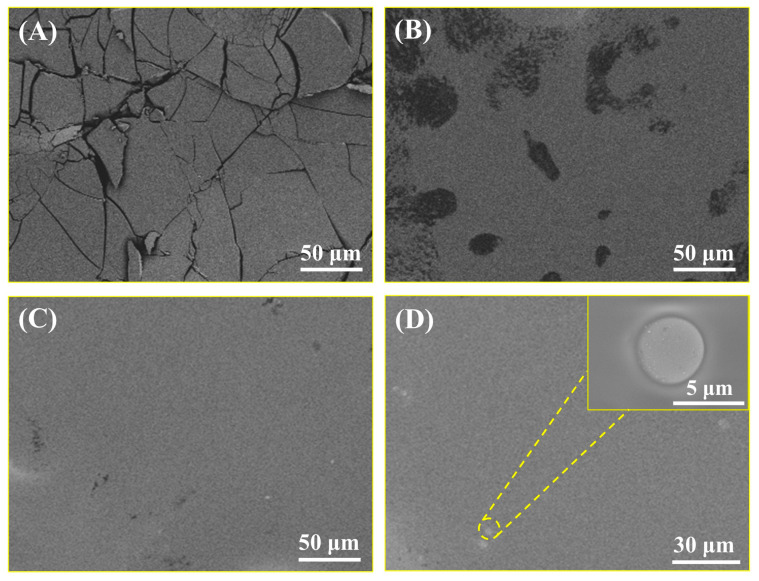
SEM images of (**A**) sample A, (**B**) sample B, (**C**) sample C, and (**D**) sample D after stretching.

Firstly, the micromorphological variation of the co-assembled sunscreen film was observed by SEM, thus qualitatively evaluating the role of each component in enhancing the toughness of the sunscreen film from the raw material level. Figure 1A shows the longitudinal and transverse cracks in the membrane layer, indicating that trimethylsiloxysilicate used as the hard film was a brittle material, which was prone to reaching failure strain upon stretching, leading to the membrane layer cracking and forming fragments. Figure 1B demonstrates that the membrane cracking of polysiloxane-15 was different from that of trimethylsiloxysilicate, showing a localized hole-type breakage (the exposed black area is the conductive adhesive substrate). The reason was that although polysiloxane-15 possessed the toughness of a flexible material, its inherent lack of strength led to holes during the stretching process. In addition, large areas of the membrane layer in Figure 1C remained basically intact with only a few smaller pores, indicating that the combination of polysiloxane-15 and trimethylsiloxysilicate significantly improved the film-forming toughness of the samples. In this case, the flexible chain segments of polysiloxane-15 could reduce the crosslinking density of the film groups, thereby increasing the free space of the siloxane chains to absorb impact energy and disperse stress [33,34]. Strikingly, after the addition of VDSC, based on the soft and hard film-compounded sample, the integrity of the film layer in Figure 1D was further enhanced compared to that in Figure 1C. No pores and cracks were found, and VDSC was observed to be uniformly dispersed and embedded in the film layer, indicating that the introduction of the functional powder further enhanced the film-forming toughness of the co-assembled sunscreen film through generating interfacial effects [35].

### 3.2. Effect of the Co-Assembled Sunscreen Film on Anti-Stretching Property

**Figure 2:**
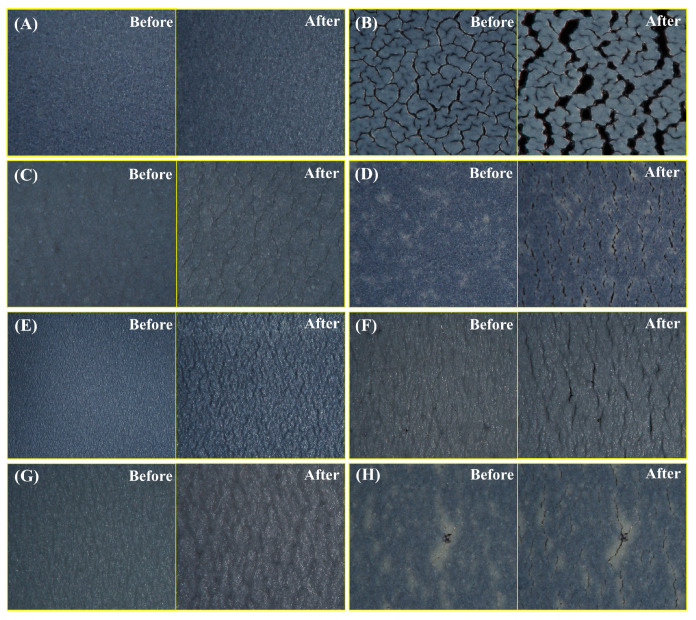
Film-forming conditions of sunscreen samples (**A**) CA, (**B**) BC, (**C**) NS, (**D**) NH, (**E**) NP, (**F**) RS, (**G**) RH, and (**H**) RP before and after stretching.

The film-forming toughness of each sunscreen formulation was reflected by tensile tests. As shown in Figure 2, different combinations of film-forming agents and powders resulted in significant differences in the film-forming toughness of the samples. Figure 2A shows that the coating of sample CA containing the co-assembled sunscreen film remained uniform and intact during the stretching process, with no holes or cracks, indicating that the co-assembled sunscreen film constructed by the specific film-forming agents and the functional powder could effectively enhance the toughness of the sunscreen product against damage induced by movement of the facial skin. Figure 2B demonstrates that the film layer of blank sample BC was already broken before stretching, and the sunscreen coating further cracked with visible fractures during stretching, which undoubtedly worsened the UV absorption effect. When the specific combination of film-forming agents and powder in the co-assembled sunscreen film was varied, the coatings of sunscreen samples were found to be broken to varying degrees, which was consistent with the results in Figure 1. Specifically, when the soft (polysiloxane-15) and hard (trimethylsiloxysilicate) films were removed, the film layers of the corresponding samples NS (Figure 2C) and NH (Figure 2D) showed holes, cracks, and other damage after stretching. Surprisingly, when the functional powder (VDSC) was removed in the co-assembled sunscreen film, the sample NP (Figure 2E) was found to exhibit small cracks during stretching as well, further confirming that the functional powder played an important role in enhancing the toughness of the sunscreen film. As the powder had a similar silicone-oxygen bonded structure as the soft and hard films, its excellent compatibility allowed it to work synergistically with the film-forming agents to jointly resist the damage to the sunscreen caused by facial skin movement.

To investigate the reasons why the components of the co-assembled sunscreen film enhanced the toughness, each component was replaced separately. Figure 2F shows the film-forming conditions of sample RS with the soft film replaced by dextrin palmitate before and after stretching, revealing that the film layer of sample RS showed obvious cracking after stretching, which was probably caused by the poor compatibility of dextrin palmitate and trimethylsiloxysilicate, resulting in insufficient binding of the film-forming agents in the formulation and a decrease in the anti-stretching property. Sample RH in Figure 2G reconfirmed that the structural similarity between the sunscreen films played a key role in enhancing the mechanical properties. When the less compatible polysiloxane-15 and VP/eicosene copolymer were combined as soft and hard films, the sunscreen film was found to have difficulty resisting external stretching, and the same cracking condition occurred. Notably, when the functional powder in the co-assembled sunscreen film was replaced with talc, which was found to exhibit poor dispersion in the system according to Figure 2H, sample RP before stretching exhibited obvious particles and powder agglomerates on the surface of the coating, and clear cracks appeared in the coating during stretching. The above results indicated that the primary reason for the excellent toughness of the co-assembled sunscreen film was the high structural similarity of the components, and the excellent compatibility allowed them to fully combine and undergo inter-component interactions.

**Figure 3:**
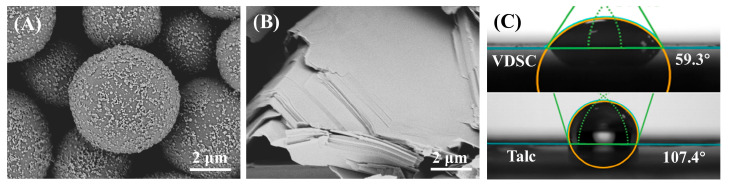
SEM images of (**A**) VDSC and (**B**) talc. (**C**) Contact angles of pure VDSC and talc to film-forming agent solution.

In addition, to reveal the strengthening mechanism of the mechanical properties of the sunscreen film by the functional powders, the microscopic morphology of different powders was analyzed by SEM and compared. The SEM images of VDSC are shown in Figure 3A and Figure S4, exhibiting ~70 nm nanoparticles attached to the surface of ~5 μm microspheres, which possessed a large specific surface area. The rough nanoparticle edges of VDSC could act as “tentacles” to grasp and embed the film layer formed by the film-forming agents, which could enhance its interfacial combination with film-forming agents through chemical bonding to realize film-forming reinforcement [35]. By contrast, the microscopic morphology of talc presented a large nano-sheet structure (Figure 3B), the highly stacked structure of which led to a substantial decrease in specific surface area as well as poor compatibility and bonding with the soft and hard films, thus worsening the film-forming toughness of the sunscreen film after replacing the VDSC with talc. Furthermore, the wettability of pure VDSC and talc to the film-forming agents was compared through contact angle tests (Figure 3C), which were 59.3° and 107.4° for VDSC and talc, respectively. The smaller contact angle was attributed to the fact that VDSC possessed a similar silicone-oxygen bonded structure as silicone-based film-forming agents, predicting high compatibility and strong interactions with the film-forming agents, which further confirmed the dominant role of structural similarity in enhancing the binding of film-forming agents and powders.

### 3.3. Effect of the Co-Assembled Sunscreen Film on Anti-Migrating Property

**Figure 4:**
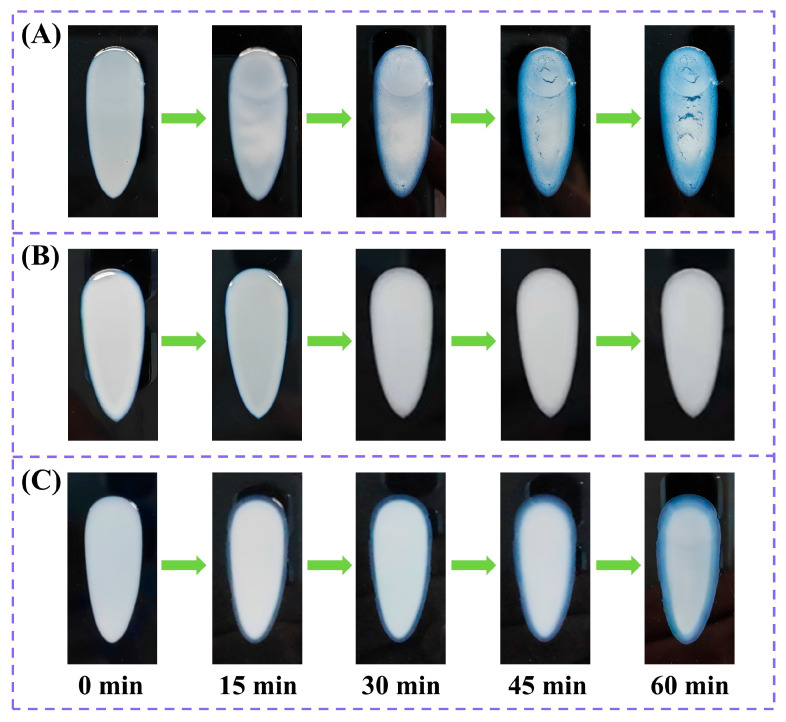
The migration conditions of coatings during drying of sunscreen samples (**A**) BC, (**B**) CA, and (**C**) NP. The time interval for each graph from left to right is 15 min.

The anti-migrating property of the co-assembled sunscreen film was next analyzed by tracking the coating distribution during the evaporation of the sunscreen formulations. As shown in Figure 4A, the blank sample BC showed agglomeration of the coating visible to the naked eye after 15 min, with non-uniform distribution of the coating and significant cracking after 45 min, which was attributed to the severe aggregation of the paste (including powders, sunscreen agents, etc.) during the evaporation process [18]. After introducing the co-assembled sunscreen film, sample CA (Figure 4B) had a uniform coating distribution throughout the volatilization process without any cracking, indicating that the presence of the co-assembled sunscreen film could effectively anchor the paste to avoid its migration and aggregation during the volatilization process, which contributed to a uniform distribution of sunscreen agents in the film layer to maximize the sunscreen effect. The anti-migrating effect of the functional powder in the co-assembled sunscreen film was also investigated, and it was found that the paste at the edge of sample NP (Figure 4C) showed obvious migration to the interior after the removal of VDSC. Therefore, the introduction of the powder could be inferred to inhibit the dehumidification of the sunscreen cream and make the sunscreen agents stable [18], facilitating the formation of a uniform sunscreen film, which proved that the film-forming agents and functional powder of the co-assembled sunscreen film exerted a full synergistic effect in terms of anti-migration.

### 3.4. Effect of the Co-Assembled Sunscreen Film on Anti-Rubbing Property

**Figure 5:**
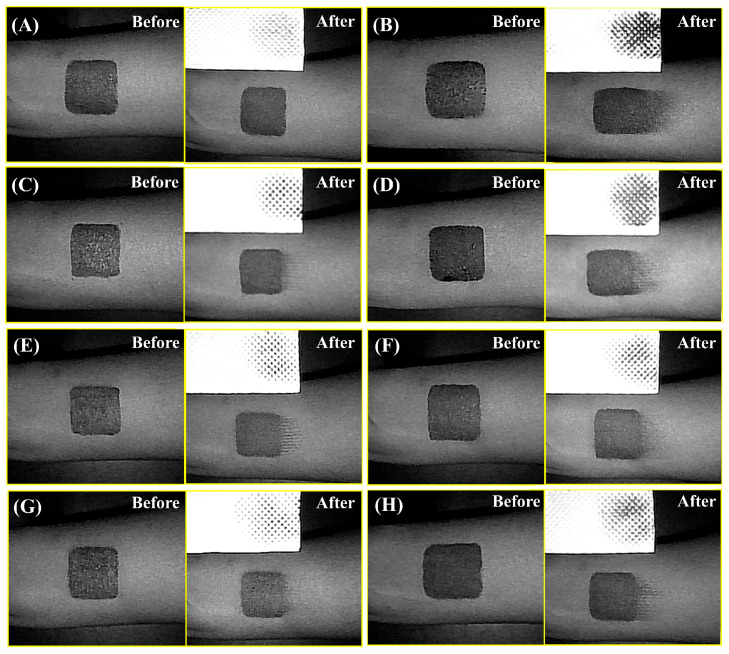
Film-forming conditions of sunscreen samples (**A**) CA, (**B**) BC, (**C**) NS, (**D**) NH, (**E**) NP, (**F**) RS, (**G**) RH, and (**H**) RP before and after rubbing.

The effectiveness of the different sunscreen formulations against external friction was evaluated by means of anti-friction tests. As shown in Figure 5, different combinations of film-forming agents and powders had a significant effect on the anti-rubbing properties of the sunscreen samples. Figure 5A presents the film-forming conditions before and after rubbing of the sample containing the co-assembled sunscreen film. Sample CA was able to greatly maintain the initial state of the film layer after the rubbing treatment, with no obvious scratches on the edges and only trace residues on the tissue paper. However, a large amount of sunscreen product was carried away when blank sample BC (Figure 5B) was subjected to friction, the coating was severely damaged, and obvious scratches appeared on the edges. The above results indicated that the interaction of film-forming agents and functional powder with similar silicone-oxygen bonded structures significantly enhanced the friction-resistant property of the sunscreens. To verify the necessity of each component in the co-assembled sunscreen film, the anti-rubbing properties of the samples after removing the soft film, hard film, and functional powder were compared, respectively. Samples NS (Figure 5C) and NH (Figure 5D) were found to have different degrees of scratches on the coating edges after rubbing, and the film layer of sample NH was more obviously broken after rubbing, which indicated that trimethylsiloxysilicate provided a better anti-rubbing effect than polysiloxane-15. When the functional powder was removed, Figure 5E shows that the membrane edges of sample NP were scratched after rubbing, and part of the sunscreen agents were removed by the paper towel, indicating that the presence of VDSC had a further reinforcing effect on the soft and hard film groups, which was consistent with the role of the functional powder in the anti-stretching and anti-migrating tests, as described previously.

To further confirm the effectiveness of structurally similar-oriented construction of sunscreen membranes, the components in the co-assembled sunscreen film were replaced by film-forming agents and powder with non-siloxane structures. Figure 5F shows the film-forming conditions of sample RS with the soft film replaced by dextrin palmitate before and after rubbing treatment, in which scratches were found to appear on the film edges after rubbing. Residual amounts of the sunscreen paste on the tissue paper were observed for samples CA and NS, indicating that dextrin palmitate could provide some anti-friction ability but was not as effective as polysiloxane-15, which was probably due to the poor compatibility between dextrin palmitate and trimethylsiloxysilicate leading to worse bonding between the soft and hard films, thus deteriorating the anti-rubbing performance of the sunscreen film. Similar results are shown in Figure 5G, where sample RH also showed scratches on the film edges after rubbing, which was attributed to the poor compatibility between VP/eicosene copolymer and polysiloxane-15, again confirming the importance of structural similarity in enhancing the mechanical properties of sunscreen films. Notably, Figure 5H demonstrates that the amount of paste removed by the tissue paper for sample RP after rubbing was significantly higher than that for sample NP, which was attributed to the poor compatibility of talc with the hard and soft film groups, which made it difficult to achieve sufficient embedding and exert a reinforcing effect on the film groups. Meanwhile, the poor dispersion of talc led to a rough and grainy film, so that the sunscreen agents were more likely to be carried away with the paste when the sample was subjected to friction, and scratches appeared on the edges. The above results indicated that the anti-friction property of the co-assembled sunscreen film mainly originated from the structural similarity of the components, which strengthened the film-forming agent–powder bonding to resist external friction.

### 3.5. FTIR and XPS Analyses

**Figure 6:**
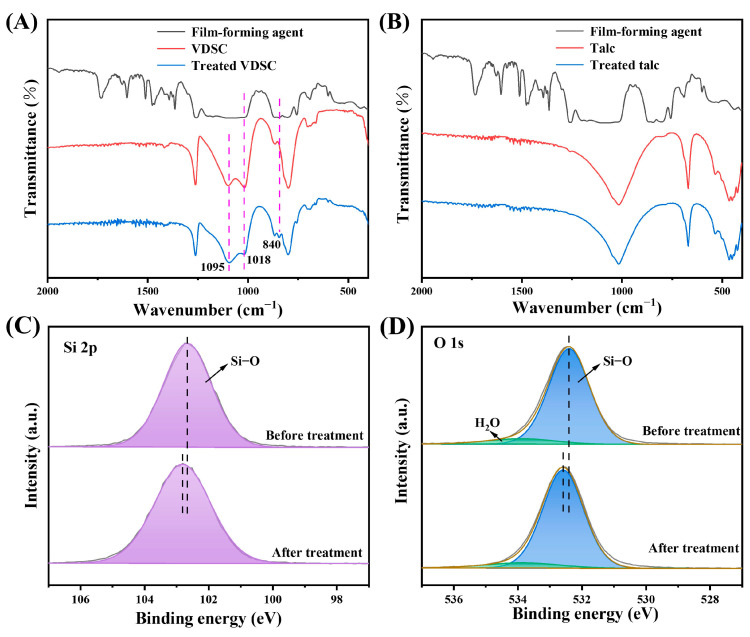
FTIR spectra of film-forming agent, (**A**) VDSC, and (**B**) talc before and after treatment. (**C**) Si 2p and (**D**) O 1s XPS spectra of VDSC before and after treatment.

The excellent performance of the co-assembled sunscreen film was mainly attributed to the film-forming agent–powder interaction, which was investigated by a combination of FTIR and XPS tests. As seen in Figure 6A, the absorption peaks (positioned by the pink dashed lines) of VDSC changed after mixed treatment with film-forming agents, the relative intensities of the Si−O stretching vibrational absorption peaks at 1018 and 1095 cm^−1^ varied [36,37], and a new vibrational absorption peak appeared at 840 cm^−1^ [38], which proved that strong interactions between VDSC and the film-forming agents occurred in the sunscreen film. By contrast, no change in absorption peaks was observed for talc with a non-silicone-oxygen bonded structure (Figure 6B), implying no intrinsic interactions between talc and film-forming agents, further confirming the specificity among the components of the co-assembled sunscreen film. In addition, the interaction between VDSC and film-forming agents was further revealed by XPS tests. Figure 6C shows the Si 2p XPS spectra of VDSC before and after treatment. The signal at 102.6 eV was assigned to the Si−O bond [39,40]. After mixed treatment with film-forming agents, the Si−O bond in VDSC shifted toward higher binding energy (positioned by the black dashed lines). The signals located at 532.4 and 533.9 eV in the O 1s XPS plot were attributed to the Si−O bond and absorbed water, respectively (Figure 6D) [41,42], demonstrating that the same binding energy enhancement occurred in the Si−O bond of VDSC after treatment, which again confirmed the existence of strong interactions between VDSC and the film-forming agents [43,44]. These results indicated that structural similarity between film-forming agents and powder was essential to generating the interaction that reinforced the sunscreen film, and that such interactions were primarily reflected in the enhancement of the Si−O bond binding energy. The research showed that when the sunscreen film generated cracks under external force, the VDSC powder could absorb a large amount of destructive energy and slow down or terminate the spread of cracks at the interface, thereby realizing a strengthening and toughening effect on the sunscreen film [45,46].

### 3.6. Effect of Co-Assembled Sunscreen Film on In Vitro Sun Protection Values

**Figure 7:**
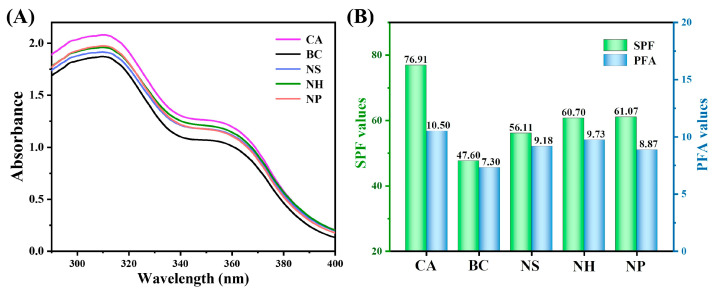
(**A**) UV absorption curves and (**B**) sun protection value test results of each sample.

To investigate the effect of the co-assembled sunscreen film on the sun protection value, UV absorption tests were performed on samples CA, BC, NS, NH, and NP, respectively. As shown in Figure 7A, sample CA exhibited the best UV absorption capacity, while the absorbance of blank sample BC was obviously weakened and showed the worst performance, which implied that the introduction of the co-assembled sunscreen film could significantly enhance the UV-shielding effect of the sunscreen formulations. Compared to the blank sample, the SPF and PFA values of sunscreen CA were increased by 61.58% and 43.84%, respectively, which demonstrated superior UV protection enhancement over other works [23,47,48]. The specific SPF and PFA values of each sample are given in Figure 7B, which showed that after removing the soft film, hard film, and functional powder in the co-assembled sunscreen film, respectively, the sun protection values of the corresponding samples NS, NH, and NP were reduced to a certain extent, suggesting that the enhanced UV absorptive capacity of the co-assembled sunscreen film originated from the synergistic interaction between film-forming agents and the powder.

**Figure 8:**
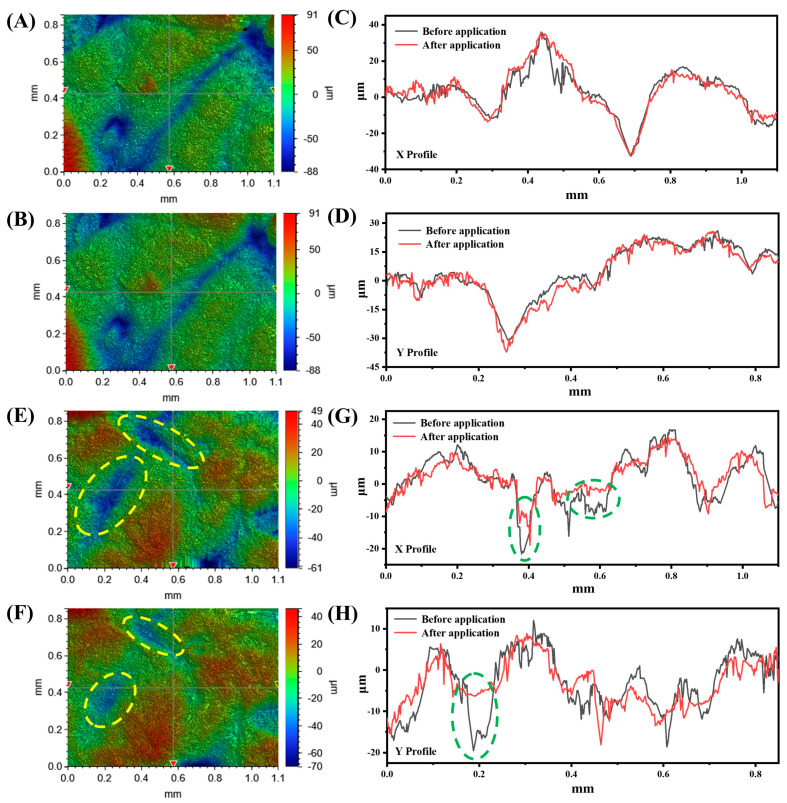
Three-dimensional contour images of pig skin (**A**) before and (**B**) after application of sunscreen CA, and contour curves of (**C**) X and (**D**) Y profiles. Three-dimensional contour images of pig skin (**E**) before and (**F**) after application of sunscreen BC, and contour curves of (**G**) X and (**H**) Y profiles.

To reveal the mechanism of the co-assembled sunscreen film to enhance the sun protection value, the film-forming conditions after the application of sunscreen samples were compared by optical profilometry. As seen in Figure 8A,B, the surface contour of the pigskin was highly consistent before and after the application of sample CA containing the co-assembled sunscreen film, and the high overlap of the X (Figure 8C) and Y (Figure 8D) profile curves reconfirmed the film-forming uniformity of sample CA. Uniform sunscreen films had been reported to maximize the effectiveness of sunscreen [25]. By contrast, the yellow dashed areas in Figure 8E,F as well as the green dashed areas in Figure 8G,H indicated that the depressions in the skin were filled with sunscreen after the application of sample BC, meaning that the sunscreen gathered in the skin grooves and formed a non-uniform film, resulting in a decrease in sun protection value. Moreover, the role of film-forming agents in the co-assembled sunscreen film was revealed by observing the film-forming conditions of the sunscreen after removing the film-forming agents. Figures S5 and S6 show that the skin-fit property of sunscreen without film-forming agents was reduced compared to sample CA, suggesting that the anti-migration effect of the film-forming agents mitigated the agglomeration and inhomogeneous distribution of the sunscreens to some extent. After removing the functional powder VDSC from the co-assembled sunscreen film, a similar decrease in film-forming uniformity was observed in Figures S7 and S8, indicating that the introduction of VDSC powder could mediate the volatilization process of the sunscreen film and allowed for uniform film formation. On the other hand, the introduction of the functional powder could enhance the solid content of the sunscreen cream, so that the film thickness after film formation increased, while relying on its diffuse reflective property through extending the optical distance to promote the value of sun protection [49].

**Figure 9:**
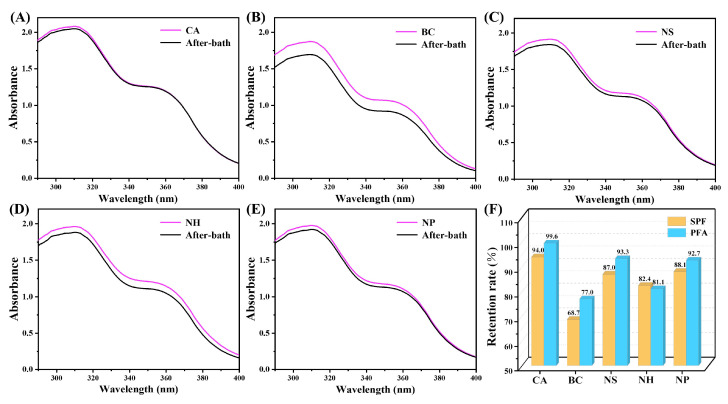
Comparison of initial and post-bath UV absorption curves of (**A**) CA, (**B**) BC, (**C**) NS, (**D**) NH, and (**E**) NP, and (**F**) sun protection value test results.

In addition, the ability of the co-assembled sunscreen film to enhance the water resistance of sunscreens was assessed by testing the retention rate of sunscreen values after bathing treatment of each sample. According to Figure 9A, the after-bath UV absorption curve of sample CA was highly consistent with the initial curve, with only a slight attenuation of UVB absorption, and the retention rates of the SPF and PFA values after bathing were 93.95 and 99.62% (Table S1), respectively. Figure 9B demonstrates the UV absorption test results of the blank sample BC, which showed that the overall absorbance after bathing exhibited obvious attenuation. The retention rates of the SPF and PFA values at this time were 68.74 and 76.99% respectively, which were significantly lower compared to sample CA, indicating that the introduction of the co-assembled sunscreen film could confer excellent water resistance to the sunscreen formulation. By analyzing the UV absorption test results of Figure 9C–E, it could be seen that the water resistance of the samples corresponding to removal of the soft film, hard film, and functional powder were all reduced compared with sample CA. The specific changes in the sun protection value are shown in Figure 9F, indicating that the enhancement of the water-resistant performance originated from the combined action of the components in the co-assembled sunscreen film, for which the hydrophobic silica-oxygen bonded structure endowed the sunscreen with excellent water resistance.

## 4. Conclusions

In summary, we constructed a co-assembled sunscreen film consisting of silicone polymer-based film-forming agents and powder with structural similarity as the guideline, which achieved the effective integration of different functional components and thus endowed the sunscreen formulation with excellent performance. The performance enhancement was mainly due to the structural similarity between the film-forming agents and functional powder, specifically, the combination of soft and hard films modulated the crosslinking density of the silicone-oxygen chains through the formation of a good interpenetrating network, thus optimizing the film strength and toughness simultaneously. The introduction of the functional powder strengthened the interfacial bonding with the film-forming agents through silicone-oxygen bonding, which further reinforced the anti-stretching, anti-migration, and anti-friction properties of the sunscreen. In addition, the film-forming agents and functional powder jointly mediated the film-forming process of the sunscreen to avoid agglomeration, resulting in a homogeneous film. Meanwhile, the introduction of the functional powder enhanced film thickness after film formation and increased contact with sunscreen agents, reflecting UV light at multiple levels, which in turn improved the sun protection property. The co-assembled sunscreen film showed an outstanding potentiation effect on sun protection values, respectively enhancing the SPF and PFA values by 61.58 and 43.84% compared with the blank sample. Benefiting from the hydrophobicity of the silicone-oxygen bonded structure, the co-assembled sunscreen film effectively enhanced the water resistance of the sunscreen formulation, with a retention rate of 93.95% for SPF and 99.62% for PFA after bathing. This work will broaden the application of polymers in cosmetics and provides a new strategy for the development of durable and efficient sunscreens.

## Figures and Tables

**Table 1:** The formulation of the sunscreen sample containing the co-assembled sunscreen film.

Phase	Component	Content (%)
A	Water	To 100
	Propanediol	5
	1,2-Hexanediol	0.4
	Citric acid	0.01
	Sodium citrate	0.15
B	Polysiloxane-15	2
	Trimethylsiloxysilicate	3
	Vinyl dimethicone/methicone silsesquioxane crosspolymer	2
	Ethylhexyl methoxycinnamate	6
	Ethylhexyl salicylate	3
	Octocrylene	3
	Titanium dioxide	4
	Zinc oxide	7
	Cyclopentasiloxane	9
	Dimethicone	8
	PEG-9 Polydimethylsiloxyethyl dimethicone	2.5
	PEG-10 Dimethicone	1.5
	Tocopheryl acetate	0.2
C	Isododecane	9
	Alcohol	8
	Phenoxyethanol	0.36
	Ethylhexylglycerin	0.04

**Table 2:** Differences in the composition of co-assembled sunscreen film in sunscreen formulations.

Component	Content (%)				
	CA	BC	NS	NH	NP
Polysiloxane-15	2	/	/	2	2
Trimethylsiloxysilicate	3	/	3	/	3
VDSC	2	/	2	2	/

## Data Availability

Data are contained within the article and the Supplementary Information.

## Supplementary Materials

The following supporting information can be downloaded at: https://www.mdpi.com/article/10.3390/polym16233317/s1, Figure S1: Structural formula of polysiloxane-15; Figure S2: Structural formula of trimethylsiloxysilicate; Figure S3: Structural formula of VDSC; Figure S4: SEM image of VDSC surface-attached nanoparticles; Figure S5: Three-dimensional contour images of pig skin (A) before and (B) after application of sunscreen without film-forming agents; Figure S6: Contour curves of (A) X and (B) Y profiles before and after application of sunscreen without film-forming agents; Figure S7: Three-dimensional contour images of pig skin (A) before and (B) after application of sunscreen without VDSC; Figure S8: Contour curves of (C) X and (D) Y profiles before and after application of sunscreen without VDSC; Table S1: Retention rate of sun protection values for each sample after water bath treatment.

## References

[1] Verma A., Zanoletti A., Kareem K.Y., Adelodun B., Kumar P., Ajibade F.O., Silva L.F.O., Phillips A.J., Kartheeswaran T., Bontempi E. (2024). Skin protection from solar ultraviolet radiation using natural compounds: A review. Environ. Chem. Lett..

[2] Byun K.-A., Lee S.Y., Oh S., Batsukh S., Jang J.-W., Lee B.-J., Rheu K.-M., Li S., Jeong M.-S., Son K.H. (2024). Fermented Fish Collagen Attenuates Melanogenesis via Decreasing UV-Induced Oxidative Stress. Mar. Drugs.

[3] Liu Y., Qin D., Wang H., Zhu Y., Bi S., Liu Y., Cheng X., Chen X. (2023). Effect and mechanism of fish scale extract natural hydrogel on skin protection and cell damage repair after UV irradiation. Colloids Surf. B Biointerfaces.

[4] Griffin G.K., Booth C.A.G., Togami K., Chung S.S., Ssozi D., Verga J.A., Bouyssou J.M., Lee Y.S., Shanmugam V., Hornick J.L. (2023). Ultraviolet radiation shapes dendritic cell leukaemia transformation in the skin. Nature.

[5] Salminen A., Kaarniranta K., Kauppinen A. (2022). Photoaging: UV radiation-induced inflammation and immunosuppression accelerate the aging process in the skin. Inflamm. Res..

[6] Lee S.C., Yoo E., Lee S.H., Won K. (2020). Preparation and application of light-colored lignin nanoparticles for broad-spectrum sunscreens. Polymers.

[7] Wang C., Wang D., Dai T., Xu P., Wu P., Zou Y., Yang P., Hu J., Li Y., Cheng Y. (2018). Skin pigmentation-inspired polydopamine sunscreens. Adv. Funct. Mater..

[8] Girard V., Fragnières L., Chapuis H., Brosse N., Marchal-Heussler L., Canilho N., Parant S., Ziegler-Devin I. (2024). The Impact of Lignin Biopolymer Sources, Isolation, and Size Reduction from the Macro-to Nanoscale on the Performances of Next-Generation Sunscreen. Polymers.

[9] Cardillo D., Sencadas V., Devers T., Islam M., Tehei M., Rosenfeld A., Boutard T., Rocher E., Barker P.J., Konstantinov K. (2021). Attenuation of UV absorption by poly (lactic acid)-iron oxide nanocomposite particles and their potential application in sunscreens. Chem. Eng. J..

[10] Lee S.J., Lee D., Park S.A., Park J.J., Park W.H. (2024). Hyaluronic acid/polyphenol sunscreens with broad-spectrum UV protection properties from tannic acid and quercetin. Int. J. Biol. Macromol..

[11] Zhang Z., Wang Y., Li T., Wu J., Huang J., Jiang J., Chen M., Dong W. (2023). Mussel-inspired anti-permeation hybrid sunscreen with reinforced UV-blocking and safety performance. Colloids Surf. A Physicochem. Eng. Aspects.

[12] van Bodegraven M., Kröger M., Zamudio Díaz D.F., Lohan S.B., Moritz R.K., Möller N., Knoblich C., Vogelsang A., Milinic Z., Hallhuber M. (2024). Redefine photoprotection: Sun protection beyond sunburn. Exp. Dermatol..

[13] Hayag M.V., Chartier T., DeVoursney J., Tie C., Machler B., Taylor J. (1997). A high SPF sunscreen’s effects on UVB-induced immunosuppression of DNCB contact hypersensitivity. J. Dermatol. Sci..

[14] Williams J.D., Maitra P., Atillasoy E., Wu M.-M., Farberg A.S., Rigel D.S. (2018). SPF 100+ sunscreen is more protective against sunburn than SPF 50+ in actual use: Results of a randomized, double-blind, split-face, natural sunlight exposure clinical trial. J. Am. Acad. Dermatol..

[15] Li H., Colantonio S., Dawson A., Lin X., Beecker J. (2019). Sunscreen application, safety, and sun protection: The evidence. J. Cutan. Med. Surg..

[16] Diffey B.L. (2001). When should sunscreen be reapplied?. J. Am. Acad. Dermatol..

[17] Keshavarzi F., Knudsen N., Brewer J.R., Ebbesen M.F., Komjani N.M., Moghaddam S.Z., Jafarzadeh S., Thormann E. (2021). In vitro skin model for characterization of sunscreen substantivity upon perspiration. Int. J. Cosmet. Sci..

[18] Ou-Yang H., Jiang L.I., Meyer K., Wang S.Q., Farberg A.S., Rigel D.S. (2017). Sun protection by beach umbrella vs sunscreen with a high sun protection factor: A randomized clinical trial. JAMA Dermatol..

[19] Ruvolo E., Aeschliman L., Cole C. (2020). Evaluation of sunscreen efficacy over time and re-application using hybrid diffuse reflectance spectroscopy. Photodermatol. Photoimmunol. Photomed..

[20] Binks B.P., Fletcher P.D., Johnson A.J., Marinopoulos I., Crowther J.M., Thompson M.A. (2016). Evaporation of particle-stabilized emulsion sunscreen films. ACS Appl. Mater. Interfaces.

[21] Giacomoni P.U., Teta L., Najdek L. (2010). Sunscreens: The impervious path from theory to practice. Photochem. Photobiol. Sci..

[22] Ngoc L.T.N., Van Tran V., Moon J.-Y., Chae M., Park D., Lee Y.-C. (2019). Recent trends of sunscreen cosmetic: An update review. Cosmetics.

[23] Infante V.H.P., Campos P.M., Calixto L., Darvin M., Kröger M., Schanzer S., Lohan S., Lademann J., Meinke M. (2021). Influence of physical–mechanical properties on SPF in sunscreen formulations on ex vivo and in vivo skin. Int. J. Pharm..

[24] Kakuda L., Campos P.M.B.G.M., Zanin R.B., Favaro L.N. (2023). Development of multifunctional sunscreens: Evaluation of physico-mechanical and film-forming properties. Int. J. Pharm..

[25] Binks B.P., Brown J., Fletcher P.D., Johnson A.J., Marinopoulos I., Crowther J.M., Thompson M.A. (2016). Evaporation of sunscreen films: How the UV protection properties change. ACS Appl. Mater. Interfaces.

[26] Keshavarzi F., Knudsen N., Komjani N.M., Ebbesen M.F., Brewer J.R., Jafarzadeh S., Thormann E. (2022). Enhancing the sweat resistance of sunscreens. Skin Res. Technol..

[27] Sohn M., Herzog B., Osterwalder U., Imanidis G. (2016). Calculation of the sun protection factor of sunscreens with different vehicles using measured film thickness distribution—Comparison with the SPF in vitro. J. Photochem. Photobiol. B Biol..

[28] Tan N.C., Djordjevic I., Malley J.A., Kwang A.L., Ikhwan S., Šolić I., Singh J., Wicaksono G., Lim S., Steele T.W. (2021). Sunlight activated film forming adhesive polymers. Biomater. Adv..

[29] Yu S., Lu Y., Guo S., Guo T., Takagi A., Kamkar M., Rojas O.J. (2023). Lignin-Polylactide Reverse Emulsions for Water and UV-Resistant Composite Films. ACS Sustain. Chem. Eng..

[30] Sohn M., Buehler T., Imanidis G. (2016). Repartition of oil miscible and water soluble UV filters in an applied sunscreen film determined by confocal Raman microspectroscopy. Photochem. Photobiol. Sci..

[31] Li P., Wang S., Zhou S. (2020). Comfortable skin sunscreens based on waterborne cross-linkable polydimethylsiloxane coatings. J. Mater. Chem. C.

[32] (2021). Cosmetics—Determination of sunscreen UVA photoprotection in vitro.

[33] Zhang Y., Yang K., Liu R., Yao J., Yan H. (2023). Superior tough, highly wear durable and self-lubricating epoxy composite co-enhanced by soft and hard nanomaterials. Chem. Eng. J..

[34] Yamawake K., Hayashi M., Nobukawa S. (2022). Preparation of All Amorphous PMMA Resins Based on the Graft Architecture with a Flexible Main Chain for Simultaneous Enhancement of Thermal and Mechanical Toughness. Macromol. Chem. Phys..

[35] Gilbert E.N., Hayes B.S., Seferis J.C. (2003). Interlayer toughened unidirectional carbon prepreg systems: Effect of preformed particle morphology. Compos. Part A Appl. Sci. Manuf..

[36] Yin Y., Yin J., Zhang W., Tian H., Hu Z., Ruan M., Xu H., Liu L., Yan X., Chen D. (2018). FT-IR and micro-Raman spectroscopic characterization of minerals in high-calcium coal ashes. J. Energy Inst..

[37] Jakobsson S. (2002). Determination of Si/Al ratios in semicrystalline aluminosilicates by FT-IR spectroscopy. Appl. Spectrosc..

[38] Yusuf M.O. (2023). Bond characterization in cementitious material binders using Fourier-transform infrared spectroscopy. Appl. Sci..

[39] Coxon P.R., Coto M., Juzeliunas E., Fray D.J. (2015). The use of electro-deoxidation in molten salts to reduce the energy consumption of solar grade silicon and increase the output of PV solar cells. Prog. Nat. Sci. Mater. Int..

[40] Kaur A., Chahal P., Hogan T. (2015). Selective fabrication of SiC/Si diodes by excimer laser under ambient conditions. IEEE Electron Device Lett..

[41] Xiao Z., Yu C., Lin X., Chen X., Zhang C., Jiang H., Zhang R., Wei F. (2020). TiO_2_ as a multifunction coating layer to enhance the electrochemical performance of SiO_x_@ TiO_2_@C composite as anode material. Nano Energy.

[42] Krishnan P., Liu M., Itty P.A., Liu Z., Rheinheimer V., Zhang M.-H., Monteiro P.J.M., Yu L.E. (2017). Characterization of photocatalytic TiO_2_ powder under varied environments using near ambient pressure X-ray photoelectron spectroscopy. Sci. Rep..

[43] Chan M.-L., Lau K.-T., Wong T., Cardona F. (2011). Interfacial bonding characteristic of nanoclay/polymer composites. Appl. Surf. Sci..

[44] Bagus P.S., Nelin C.J., Brundle C.R. (2023). Chemical significance of x-ray photoelectron spectroscopy binding energy shifts: A Perspective. J. Vac. Sci. Technol. A.

[45] Shin H., Kim B., Han J.-G., Lee M.Y., Park J.K., Cho M. (2017). Fracture toughness enhancement of thermoplastic/epoxy blends by the plastic yield of toughening agents: A multiscale analysis. Compos. Sci. Technol..

[46] Picu C.R., Krawczyk K.K., Wang Z., Pishvazadeh-Moghaddam H., Sieberer M., Lassnig A., Kern W., Hadar A., Constantinescu D.M. (2019). Toughening in nanosilica-reinforced epoxy with tunable filler-matrix interface properties. Compos. Sci. Technol..

[47] Peres D.D., Sarruf F.D., de Oliveira C.A., Velasco M.V.R., Baby A.R. (2018). Ferulic acid photoprotective properties in association with UV filters: Multifunctional sunscreen with improved SPF and UVA-PF. J. Photochem. Photobiol. B Biol..

[48] Meaudre H., Aubrun O., Boitte J., Douezan S., Josso M., Le Verge D., Renoux P., Rondepierre G. (2023). New formulation technology to boost sun protection. Int. J. Cosmet. Sci..

[49] Oshina I., Spigulis J. (2021). Beer–Lambert law for optical tissue diagnostics: Current state of the art and the main limitations. J. Biomed. Opt..

