# Supplementary material for: Structural Similarity-Induced Inter-Component Interaction in Silicone Polymer-Based Composite Sunscreen Film for Enhanced UV Protection
Source: Polymers (Basel). 2024 Nov 27;16(23):3317. doi: 10.3390/polym16233317 (PMC11644281; doi:10.3390/polym16233317)
Supplement: Supplementary file 1 [file polymers-16-03317-s001.zip › polymers-3303457-supplementary.pdf]

Structural Similarity-Induced Inter-Component Interaction in Silicone Polymer-Based Composite Sunscreen Film for Enhanced UV Protection

Yuyan Chen [1], Hanwen Xu [1,*], Yuhang Liu [1], Qiuting Fu [1], Pingling Zhang [1], Jie Zhou [1], Hongyu Dong [1] and Xiaodong Yan [2,*]

1 Research & Innovation Center, Proya Cosmetics Co., Ltd., Hangzhou 310023, China
2 Key Laboratory of Synthetic and Biological Colloids, Ministry of Education, School of Chemical & Material Engineering, Jiangnan University, Wuxi 214122, China
* Correspondence: xuhanwen48@proya.com (H.X.); xiaodong.yan@jiangnan.edu.cn (X.Y.)

Table S1. Retention rate of sun protection values for each sample after water bath treatment.

Test Group	CA		BC		NS		NH		NP	
	SPF	PFA	SPF	PFA	SPF	PFA	SPF	PFA	SPF	PFA
**Initial value**	76.91	10.50	47.60	7.30	56.11	9.18	60.70	9.73	61.07	8.87
**After-bath value**	72.26	10.46	32.72	5.62	48.80	8.56	50.04	7.89	53.82	8.22
**Retention rate (%)**	93.95	99.62	68.74	76.99	86.97	93.25	82.44	81.09	88.13	92.67

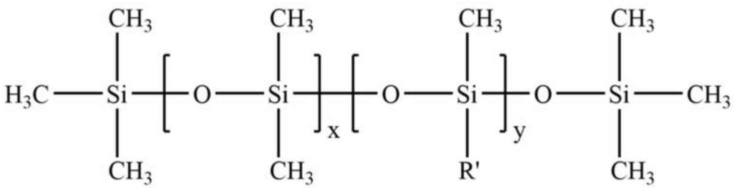

wherein
R' represents the UV absorbing groups introduced into the structure

Figure S1. Structural formula of polysiloxane-15.

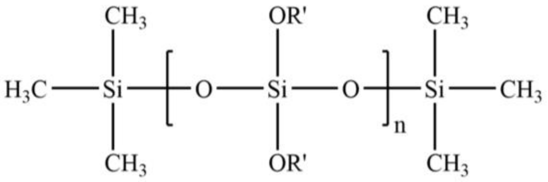

wherein
R' represents the linkage with other siloxane backbones

Figure S2. Structural formula of trimethylsiloxysilicate.

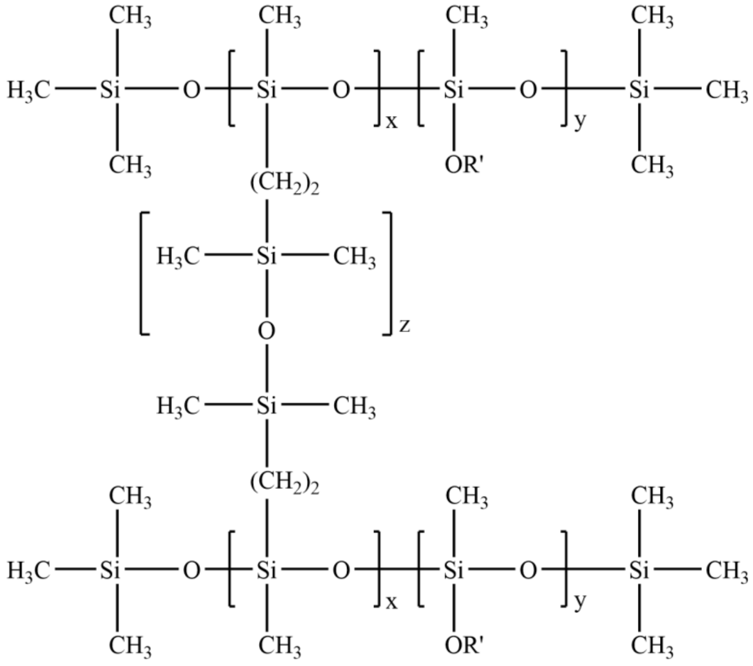

wherein
R' represents the linkage with other siloxane backbones

Figure S3. Structural formula of VDSC.

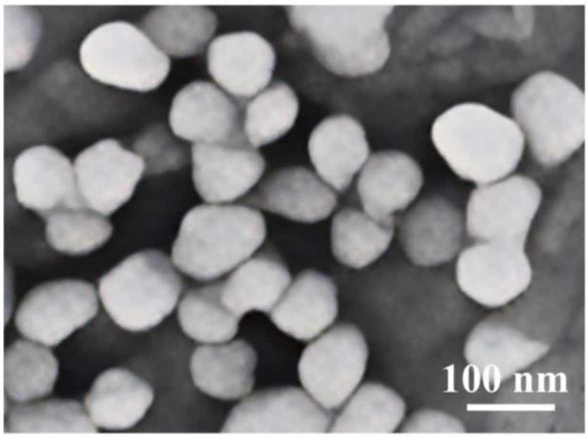

Figure S4. SEM image of VDSC surface-attached nanoparticles.

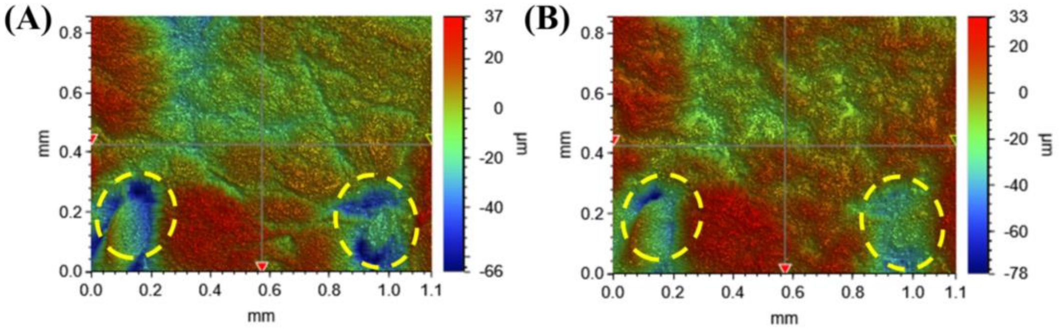

Figure S5. Three-dimensional contour images of pig skin (A) before and (B) after application of sunscreen without film-forming agents.

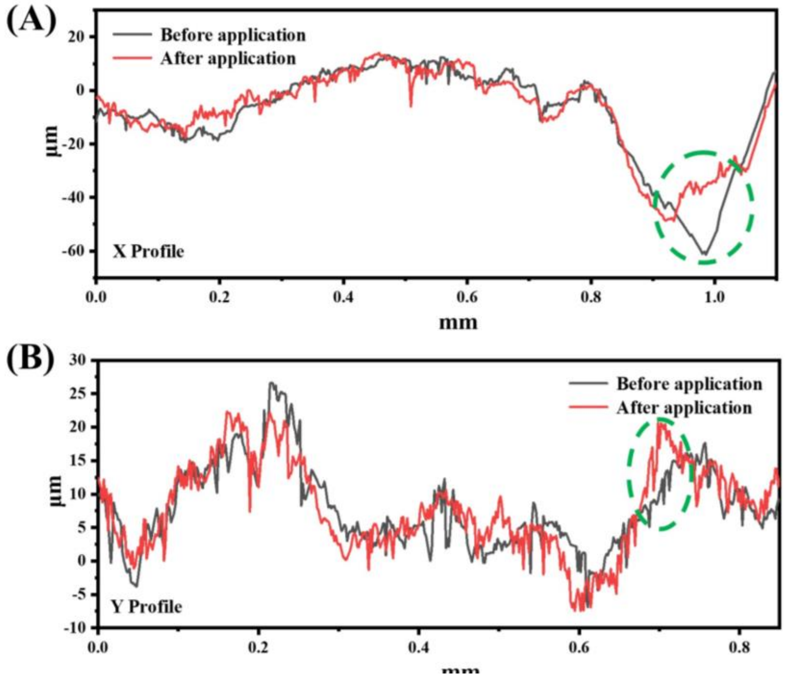

Figure S6. Contour curves of (A) X and (B) Y profiles before and after application of sunscreen without film-forming agents.

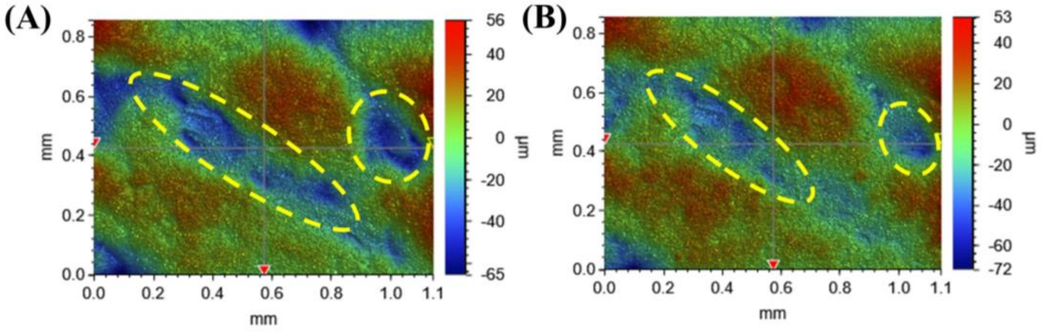

Figure S7. Three-dimensional contour images of pig skin (A) before and (B) after application of sunscreen without VDSC.

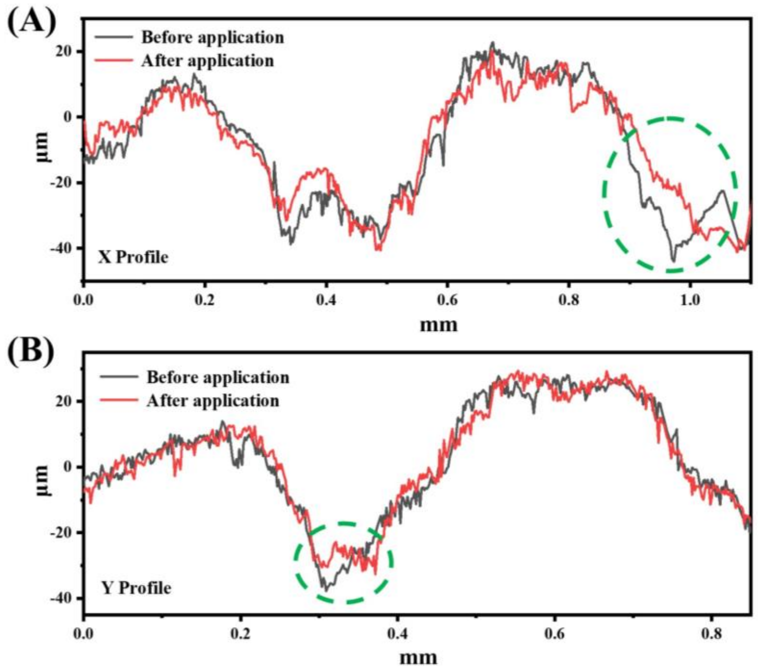

Figure S8. Contour curves of (C) X and (D) Y profiles before and after application of sunscreen without VDSC.
